# Using blood routine indicators to establish a machine learning model for predicting liver fibrosis in patients with *Schistosoma japonicum*

**DOI:** 10.1038/s41598-024-62521-1

**Published:** 2024-05-20

**Authors:** Yang Liu, Shudong Xie, Jie Zhou, Yu Cai, Pengpeng Zhang, Junhui Li, Yingzi Ming

**Affiliations:** 1 Transplantation Center, The Third Xiangya Hospital, Central South University, No. 138 Tongzipo Road, Changsha, 410013 Hunan China; 2 Engineering and Technology Research Center for Transplantation Medicine of National Health Comission, Changsha, Hunan China; 3 Hunan Province Clinical Research Center for Infectious Diseases, Changsha, Hunan China; 4 Hunan Institute of Schistosomiasis Control, Yueyang, Hunan China

**Keywords:** Immunology, Infectious diseases, Parasitic infection

## Abstract

This study intends to use the basic information and blood routine of schistosomiasis patients to establish a machine learning model for predicting liver fibrosis. We collected medical records of *Schistosoma japonicum* patients admitted to a hospital in China from June 2019 to June 2022. The method was to screen out the key variables and six different machine learning algorithms were used to establish prediction models. Finally, the optimal model was compared based on AUC, specificity, sensitivity and other indicators for further modeling. The interpretation of the model was shown by using the SHAP package. A total of 1049 patients’ medical records were collected, and 10 key variables were screened for modeling using lasso method, including red cell distribution width-standard deviation (RDW-SD), Mean corpuscular hemoglobin concentration (MCHC), Mean corpuscular volume (MCV), hematocrit (HCT), Red blood cells, Eosinophils, Monocytes, Lymphocytes, Neutrophils, Age. Among the 6 different machine learning algorithms, LightGBM performed the best, and its AUCs in the training set and validation set were 1 and 0.818, respectively. This study established a machine learning model for predicting liver fibrosis in patients with *Schistosoma japonicum*. The model could help improve the early diagnosis and provide early intervention for schistosomiasis patients with liver fibrosis.

## Introduction

*Schistosomiasis japonicum* is an infectious parasitic disease with serious consequences, widely distributed in tropical and subtropical regions of Asia, Africa and other continents^1^. According to WHO reports, schistosomiasis is still spreading and prevalent in 52 countries, affecting the health and quality of life of millions of people^2^. The cercariae of *Schistosoma japonicum* penetrate the human skin and enter the liver through the blood circulation, and then spawn in large numbers. Inflammatory granulomas form around schistosome eggs, and liver fibrosis develops gradually around this focus^3^. If patients are not treated in time, they are more likely to experience the serious consequences of cirrhosis when combined with other liver diseases. Early clinical diagnosis and treatment can increase the degree of improvement of liver fibrosis in schistosomiasis. In order to improve the quality of life and effectively reduce the risks of liver cirrhosis, peritoneal effusion, and liver cancer, early prediction and diagnosis of liver fibrosis has become an important problem to be solved in the field of diagnosis and treatment of liver fibrosis in schistosomiasis. At present, serological biomarkers and transient elastography are widely accepted clinically as the main basis for the diagnosis of early liver fibrosis^4^. But both have the same problem, that is, it is difficult to accurately diagnose liver fibrosis in stages. The stability of transient elastography measurements is easily disturbed by sampling errors, differences in instrument use, and other factors, which have certain clinical limitations^5^.

Machine learning is an artificial intelligence method used to process large amounts of complex and multi-type data, and it has achieved breakthroughs in the application of complex medical problems^6^. If the advantages of machine learning methods in describing complex data structures can be used, the degree of development of liver fibrosis in schistosomiasis can be accurately predicted and diagnosed. It can provide valuable early evidence for clinical treatment, thereby improving the quality of life and prognosis of patients.

The purpose of this study is to determine the influencing factors of liver fibrosis in schistosomiasis, based on the data of blood routine examination, to establish a machine learning model for early prediction of liver fibrosis in schistosomiasis.

## Results

### Baseline information

This study included 1049 patients, and the baseline table of the total population is shown in Table 1. The median age was 62.0 years (range 51.0–71.0). In the whole population, 281 patients (26.79%) had significant liver fibrosis, and 768 patients (73.21%) had no significant liver fibrosis.

**Table 1:** Baseline.

Variable, median (IQR)	All (n = 1049)	Non-fibrosis group (n = 768)	Fibrosis group (n = 281)	*P*-value
Age (years)	62 (51, 71)	59 (49, 69)	67 (58, 73)	< 0.001
Sex, n (%)				0.471
Female	344 (32.80)	247 (32.16)	97 (34.52)	
Male	705 (67.81)	521 (67.84)	184 (65.48)	
TT (s)	18.766 (17.901, 19.006)	18.756 (17.869, 18.766)	18.766 (18.100, 19.600)	< 0.001
Fibrinogen (g/L)	2.492 (2.399, 2.906)	2.535 (2.492, 2.964)	2.492 (2.070, 2.731)	< 0.001
Prothrombin activity (%)	133.57 (116.23, 144.50)	138.90 (120.81, 144.50)	117.46 (87.90, 142.03)	< 0.001
INR	1.057 (1.052, 1.057)	1.056 (1.053, 1.057)	1.057 (1.050, 1.160)	< 0.001
PT (s)	11.591 (11.529, 11.593)	11.589 (11.531, 11.591)	11.591 (11.500, 12.700)	< 0.001
APTT (s)	25.130 (22.710, 25.437)	24.300 (22.703,25.437)	25.437 (22.900, 28.100)	< 0.001
Glucose (mmol/L)	5.320 (4.940, 5.850)	5.340 (4.950, 5.820)	5.280 (4.920, 5.940)	0.992
LDL (mmol/L)	3.216 (2.590, 3.870)	3.310 (2.720, 3.950)	2.900 (2.240, 3.430)	< 0.001
HDL (mmol/L)	1.330 (1.120, 1.590)	1.320 (1.100, 1.560)	1.380 (1.150, 1.650)	0.005
Total cholesterol (mmol/L)	4.850 (4.220, 5.540)	4.951 (4.320, 5.670)	4.600 (3.961, 5.260)	< 0.001
Triglycerides (mmol/L)	1.180 (0.860, 1.690)	1.260 (0.930, 1.820)	0.970 (0.700, 1.340)	< 0.001
Uric acid (μmol/L), mean ± SD	349.532 ± 96.554	346.677 ± 89.926	357.417 ± 112.477	0.154
Creatinine (μmol/L), mean ± SD	70.923 ± 33.009	68.948 ± 23.433	76.359 ± 50.364	0.019
Urea nitrogen (mmol/L), mean ± SD	5.354 ± 4.229	5.185 ± 3.279	5.820 ± 6.099	0.100
Glutamyl transpeptidase (U/L)	29.000 (19.000, 53.000)	26.000 (18.000, 41.000)	52.000 (27.000, 103.000)	< 0.001
Alkaline phosphatase (U/L)	74.000 (60.000, 91.000)	70.000 (58.000, 84.000)	85.000 (69.000, 121.000)	< 0.001
Total bile acid (μmol/L)	3.000 (2.570, 6.200)	2.570 (2.400, 4.700)	6.000 (2.900, 13.900)	< 0.001
AST (U/L)	24.000 (20.000, 31.000)	22.000 (18.000, 26.000)	33.000 (28.000, 45.000)	< 0.001
ALT (U/L)	24.000 (18.000, 32.000)	21.000 (16.000, 27.000)	32.000 (25.000, 43.000)	< 0.001
Globulin (g/L)	26.000 (23.800, 28.700)	25.600 (23.700, 28.000)	26.900 (24.400, 32.000)	< 0.001
Albumin (g/L)	44.000 (40.600, 46.100)	44.800 (41.500, 46.300)	41.800 (36.200, 45.700)	< 0.001
Indirect bilirubin (μmol/L)	9.300 (7.000, 12.500)	8.800 (6.700, 11.500)	11.500 (8.200, 14.100)	< 0.001
Platelet distribution width (%)	16.000 (15.872, 16.500)	15.900 (15.872, 16.300)	16.300 (15.872, 16.900)	< 0.001
Plateletcrit (ng/mL)	0.210 (0.166, 0.218)	0.210 (0.190, 0.230)	0.148 (0.110, 0.193)	< 0.001
Platelets (*10^9^/L)	175.000 (137.000, 216.000)	197.000 (163.000, 227.000)	109.000 (79.000, 140.000)	< 0.001
RDW-CV (%)	13.300 (12.500, 13.802)	13.200 (12.400, 13.802)	13.600 (12.900, 14.311)	< 0.001
RDW-SD (%)	44.000 (42.170, 46.300)	43.600 (41.747, 45.533)	46.000 (43.300, 49.900)	< 0.001
MCHC (g/L)	328.000 (321.000, 334.000)	328.000 (321.000, 334.000)	327.000 (318.000, 334.000)	0.388
MCH (pg)	30.900 (29.800, 32.100)	30.800 (29.800, 31.800)	31.500 (29.900, 32.700)	< 0.001
MCV (fl)	94.500 (90.600, 98.200)	94.000 (90.600, 97.400)	96.000 (90.900, 99.600)	< 0.001
HCT (%)	41.933 (38.725, 44.600)	42.500 (39.900, 45.000)	39.000 (34.500, 43.100)	< 0.001
HB (g/L)	138.000 (125.000, 149.000)	140.000 (130.000, 150.000)	126.000 (111.000, 142.000)	< 0.001
Red blood cells (*10^12^/L)	4.480 (4.070, 4.840)	4.580 (4.240, 4.920)	4.110 (3.560, 4.590)	< 0.001
Basophils (*10^9^/L)	0.010 (0.010, 0.020)	0.010 (0.010, 0.020)	0.010 (0.010, 0.020)	< 0.001
Eosinophils (*10^9^/L)	0.130 (0.080, 0.210)	0.140 (0.090, 0.220)	0.120 (0.070, 0.180)	< 0.001
Monocytes (*10^9^/L)	0.320 (0.250, 0.400)	0.330 (0.260, 0.400)	0.270 (0.210, 0.370)	< 0.001
Lymphocytes (*10^9^/L)	1.650 (1.240, 2.070)	1.730 (1.380, 2.110)	1.250 (0.850, 1.790)	< 0.001
Neutrophils (*10^9^/L)	3.120 (2.430, 3.960)	3.300 (2.680, 4.190)	2.430 (1.850, 3.260)	< 0.001
White blood cell (*10^9^/L)	5.400 (4.400, 6.430)	5.610 (4.850, 6.690)	4.290 (3.340, 5.470)	< 0.001

*TT* thrombin time, *INR* international normalized ratio, *PT* prothrombin time, *APTT* activated partial thromboplastin time, *LDL* low-density lipoprotein, *HDL* high-density lipoprotein, *AST* aspartate aminotransferase, *ALT* alanine aminotransferase, *RDW*-*CV* red cell distribution width-coefficient of variation count, *RDW-SD* red cell distribution width-standard deviation, *MCHC* mean corpuscular hemoglobin concentration, *MCH* mean corpuscular hemoglobin, *MCV* mean corpuscular volume, *HCT* hematocrit, *HB* hemoglobin.

### Variable screening

A total of 10 key factors were selected by the LassoCV method: ‘RDW-SD’, ‘MCHC’, ‘MCV’, ‘HCT’, ‘Red blood cells’, ‘Eosinophils’, ‘Monocytes’, ‘Lymphocytes’, ‘Neutrophils’, ‘Age’.

### Multi-algorithm model comparison

**Figure 1:**
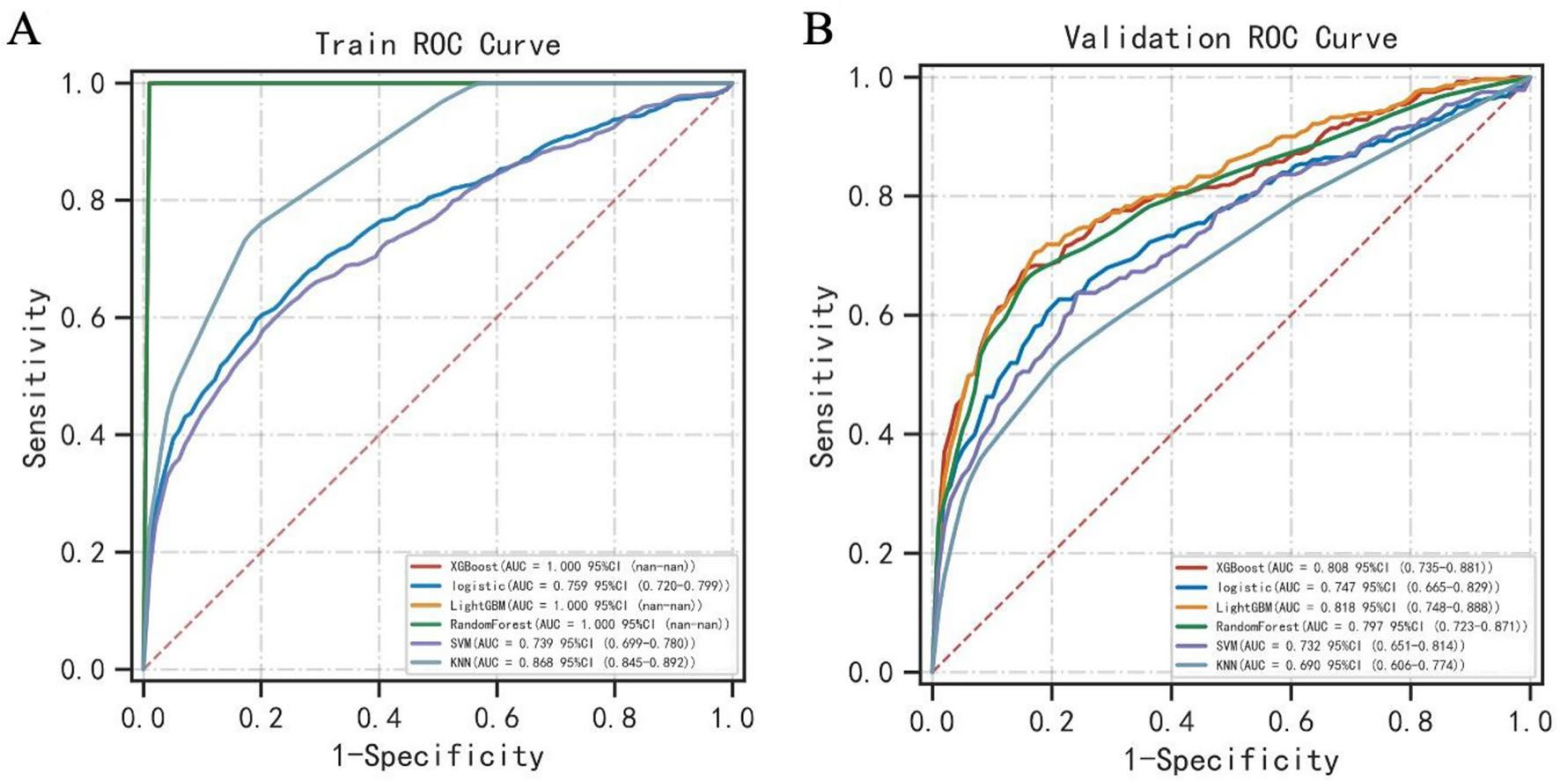
Multi-model comparison diagram. (**A**) Figure A shows the AUC of multiple models in the training set. Each color represents a machine learning algorithm. (**B**) Figure B shows the AUC of multiple models in the validation set.

Using 6 machine learning model algorithms for classification, among the 6 different machine learning algorithms, LightGBM performed the best, and its AUCs in the training set and validation set were 1 and 0.818, respectively (Fig. 1A,B). At the same time, its cutoff value, accuracy, sensitivity, specificity, positive predictive value, negative predictive value, F1 score, and Kappa value are 0.876, 0.807, 0.709, 0.842, 0.842, 0.803, 0.769, and 0.394, respectively. The evaluation results of other machine learning algorithms are shown in Table 2 and Supplementary Table 1. The forest plot in Supplementary Fig. 1 shows the ROC results of each model, and the error bar in the figure is the SD of the ROC mean. The clinical decision curve in Supplementary Fig. 2 shows the LightGBM performs well and is more stable.

**Table 2:** Multi-model classification—validation set results.

Model	AUC (SD)	Cut-off (SD)	Accuracy (SD)	Sensitivity (SD)	Specificity (SD)	Positive predictive value (SD)	Negative predictive value (SD)	F1 score (SD)	Kappa (SD)
XGBoost	0.808 (0.022)	0.863 (0.011)	0.817 (0.020)	0.680 (0.045)	0.865 (0.018)	0.836 (0.064)	0.814 (0.015)	0.748 (0.044)	0.437 (0.071)
Logistic	0.747 (0.041)	0.328 (0.031)	0.767 (0.027)	0.609 (0.075)	0.832 (0.043)	0.574 (0.055)	0.844 (0.018)	0.586 (0.036)	0.410 (0.048)
LightGBM	0.818 (0.022)	0.876 (0.009)	0.807 (0.022)	0.709 (0.070)	0.842 (0.017)	0.842 (0.071)	0.803 (0.017)	0.769 (0.064)	0.394 (0.081)
RandomForest	0.797 (0.022)	0.450 (0.032)	0.805 (0.018)	0.683 (0.057)	0.827 (0.030)	0.680 (0.050)	0.838 (0.009)	0.681 (0.052)	0.463 (0.040)
SVM	0.732 (0.047)	0.273 (0.014)	0.713 (0.036)	0.620 (0.077)	0.792 (0.049)	0.475 (0.050)	0.833 (0.021)	0.537 (0.060)	0.321 (0.071)
KNN	0.690 (0.030)	0.400 (0.000)	0.776 (0.013)	0.474 (0.135)	0.840 (0.081)	0.662 (0.046)	0.795 (0.010)	0.542 (0.103)	0.324 (0.044)

### Best model

**Figure 2:**
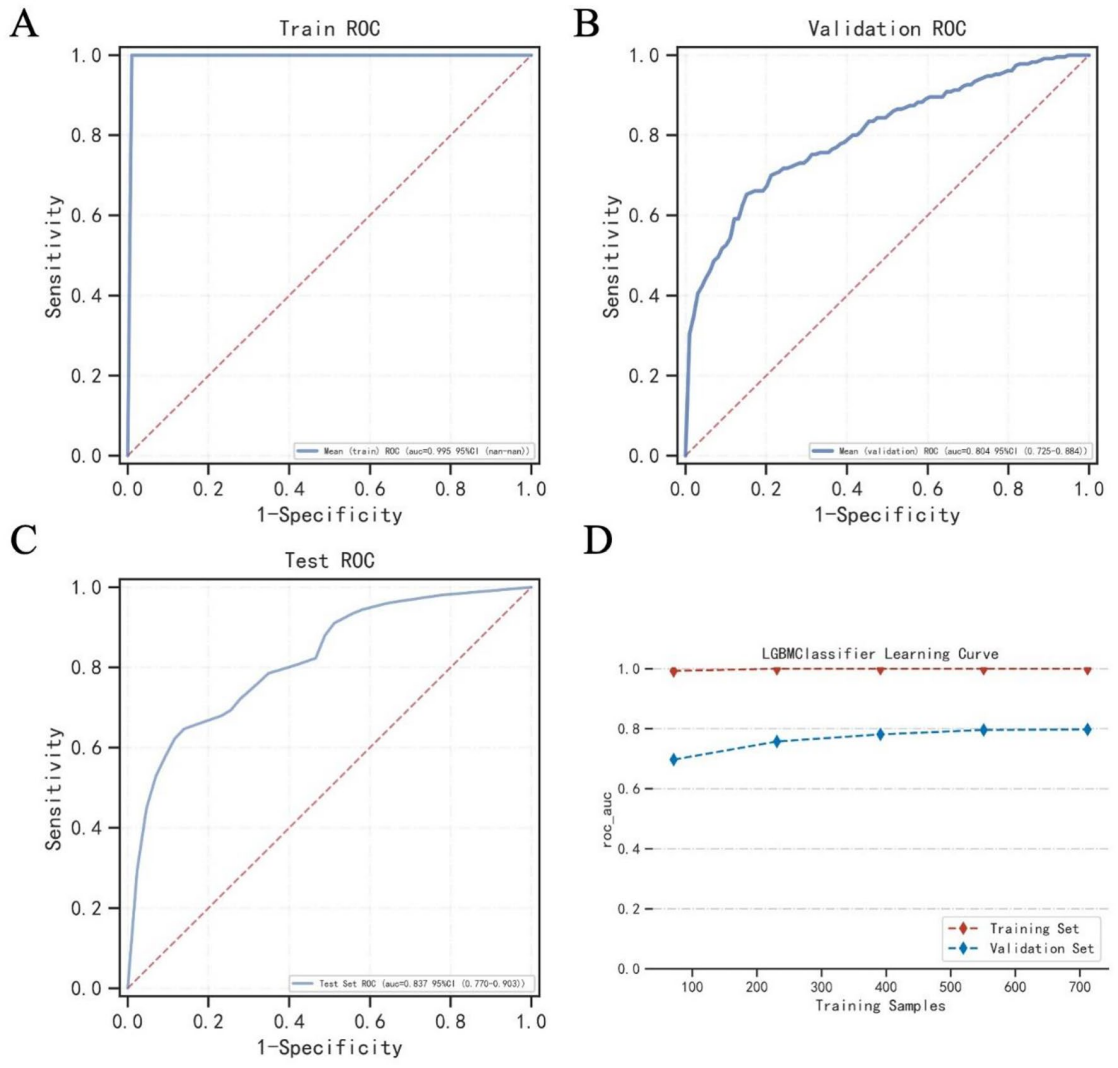
AUC of the LightGBM model. (**A**) AUC of the LightGBM model in the train set. (**B**) AUC of the LightGBM model in the validation set. (**C**) AUC of the LightGBM model in the test set. (**D**) Figure shows that the AUC of the LightGBM model changes according to the training sample size. The abscissa represents the sample number, and the ordinate represents the ROC value.

After comparing multiple models, it was found that LightGBM performed best, and we used LightGBM for modeling. The AUC in the training set was 0.995, the AUC in the validation set was 0.804, and the AUC in the test set was 0.8367 (Fig. 2A–C). At the same time, we can see that during cross-validation, when the sample size of the training set and the validation set reaches 400, the model reaches a stable state (Fig. 2D). Supplementary Tables 2–4 showed the metrics for model evaluation on the training set, validation set, and test set, respectively.

### Model interpretability

**Figure 3:**
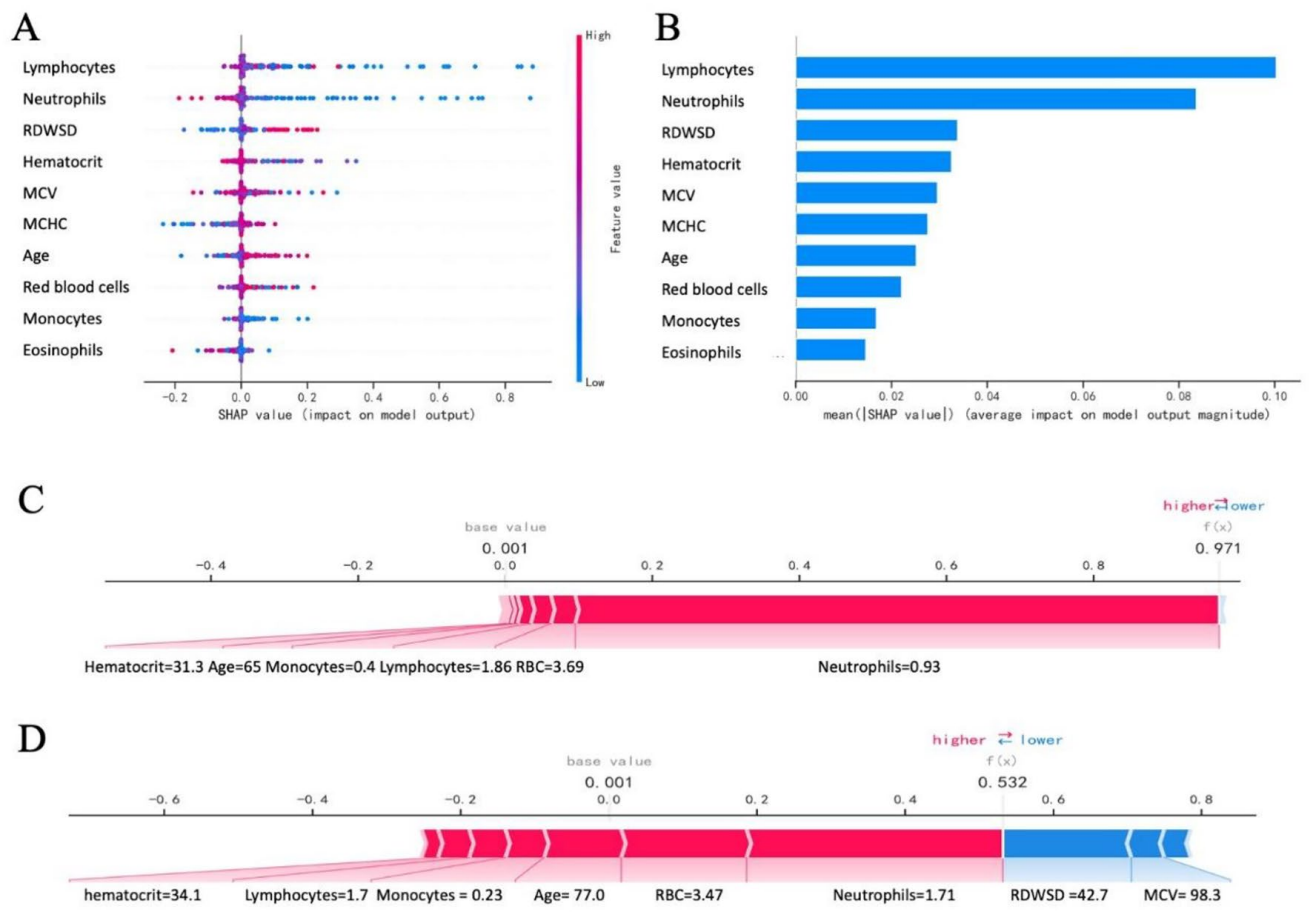
Interpretability of the model. (**A**) SHAP diagram. Each point represents a sample. The redder the color of the point, the larger the value of the variable, and the bluer the red, the smaller the value of the variable. The larger the ordinate of the point, the more likely the outcome is to be positive. (**B**) Importance ranking of key variables. The abscissa is the absolute value of the SHAP value, and the ordinate is the key variable. (**C**) The samples with a positive outcome. Red indicates a positive contribution to a positive outcome, and blue indicates a negative contribution to a positive outcome. The length of the bar indicates the size of the contribution. The longer the bar, the greater the contribution to the outcome. (**D**) The samples with negative outcome.

The SHAP diagram in Fig. 3A showed how each variable in the validation set contributes to the prediction of infection. The redder each point means that the absolute value of the point is larger, and the bluer the point, the smaller the absolute value of the point. The ordinate is a negative absolute value The larger the value, the greater the possibility of the predicted result being negative, and the greater the absolute value of the positive number on the vertical axis, the greater the possibility of the predicted result being positive. For example, the larger the RDW-SD value, the greater the possibility of liver fibrosis in patients, and the lower the possibility of liver fibrosis in patients with higher lymphocyte and neutrophil counts. Figure 3B showed the importance ranking of each variable. We can see that RDW-SD, lymphocytes and neutrophils are more important variables. Figure 3C and Fig. 3D used two force diagrams to show how the variables of the two samples affect the results. As shown in Fig. 3C, the patient was predicted to be infected, but was actually infected. We can see that the longest red arrow is neutrophils (0.93), indicating that neutrophils are the most important for the patient’s infection. The outcome had the largest positive contribution, and the second largest positive contribution was red blood cells (3.69). There were no variables that had a negative contribution to the outcome. In Fig. 3D, the patient was predicted not to have an infection, but in fact no infection occurred. The three variables that had the most positive impact were the number of neutrophils (1.71), red blood cells (3.47), and age (77.0), the two variables that had the most negative impact on the outcome were RDW-SD (42.7) and MCV (98.3).

## Discussion

After infecting the host, *Schistosoma japonicum* produces a large number of eggs and deposits them in tissues such as the liver. If timely and effective intervention is not performed, changes such as egg granuloma and liver fibrosis may further develop into hepatocellular carcinoma^7^. Studies have shown that liver fibrosis is not a single irreversible progression, and liver fibrosis may have the potential to regress^8^. Therefore, it has positive significance in the early diagnosis and treatment of liver fibrosis. At present, schistosomiasis has not attracted enough attention in major endemic countries, resulting in relatively lagging clinical and basic research on schistosomiasis, and there are few basic data research on schistosomiasis liver fibrosis^9^. This study predicts the risk of liver fibrosis by constructing a diagnostic model, which has important clinical significance for early and correct treatment and intervention.

This study uses a machine learning model to predict liver fibrosis in *Schistosomiasis japonicum*, helping clinicians to deeply understand the impact of key factors on liver fibrosis. It is helpful for early identification of liver fibrosis and distinguishing the severity of liver fibrosis, so as to timely detect patients with early liver fibrosis and improve the prognosis of them. In this study, the data of 1049 patients with *Schistosomiasis japonicum* were analyzed to establish a liver fibrosis prediction model using machine learning algorithms to help identify patients at high risk of liver fibrosis. The model established in this study is well discriminative and exhibits satisfactory specificity and sensitivity.

After screening out 10 key factors, the research uses 6 different machine learning algorithms to classify. Compared with other models, the LightGBM algorithm has better performance and higher stability, and the AUC of the optimal model is 0.8367. In the evaluation of the importance of model variables, the top three indicators with positive contribution to the outcome of liver fibrosis are neutrophils, red blood cells, and age, while the indicators with the largest negative contributions are RDW-SD and MCV. Except for the patient’s age, other indicators are related to blood routine.

Overall, the key variables included in the model may play an important role in the early diagnosis of *Schistosoma japonicum* liver fibrosis. Previous reports point out that there is an inseparable relationship between blood routine indicators and liver fibrosis^10^, and the results of this study also support this association. The neutrophil-to-lymphocyte ratio (NLR) is widely used to assess inflammatory diseases. The study found that for patients with nonalcoholic fatty liver disease (NAFLD), NLR was significantly correlated with liver fibrosis stage and nonalcoholic fatty liver disease activity score (NAS); For chronic hepatitis B (CHB) patients, NLR was negatively correlated with liver fibrosis stage^11–14^. Therefore, NLR may be associated with the stage of liver fibrosis. Kekilli et al. also demonstrated that the ratio of neutrophils to lymphocytes reflects the severity of advanced liver fibrosis^15^. RDW is a parameter reflecting the heterogeneity of red blood cell volume, which is often used to diagnose different types of anemia, and is closely related to the body’s inflammation and nutritional status. Elevated RDW often indicates shortened lifespan and increased destruction of red blood cells. Michalak et al. believe that RDW and its derivatives may be related to the deterioration of liver function^16^. Studies have shown that RDW is closely related to liver fibrosis in diseases such as NAFLD and CHB^17–19^. RDW can be expressed as RDW-CV and RDW-SD. RDW-SD is determined by the width of the red blood cell volume distribution curve above 20% above baseline. Studies have shown^20^ that RDW-SD is closely related to significant liver fibrosis (F2–F4) in CHB and can be used as an effective predictor for significant liver fibrosis in CHB. Liu et al.^21–23^ also found that only RDW-SD had a statistically significant difference between different stages of liver fibrosis in AIH (P = 0.046). In univariate Logistic regression analysis, RDW-SD was a risk factor for advanced liver fibrosis (F3–F4) in AIH. MCV is a parameter that reflects the volume of red blood cells, and changes in MCV suggest that the patient’s hemoglobin synthesis is impaired. Liu et al.^21^ further found that MCV had statistically significant differences among different stages of liver fibrosis in AIH and was positively correlated with the severity of liver fibrosis. The combination of MCV and RDW can comprehensively reflect the discrete state of peripheral red blood cell volume. So far, the mechanism between RDW, MCV and liver fibrosis is unclear, and may include the following points: (1). Inflammatory cytokines may inhibit the maturation of red blood cells and accelerate the entry of newer and larger reticulocytes into the peripheral circulation, resulting in increased RDW; (2). Patients with liver disease often have decreased intestinal absorption function, resulting in folic acid, vitamin B12 and other deficiencies, resulting in varying degrees of megaloblastic anemia and heterogeneous changes in red blood cell volume; (3). Hepatic fibrosis often causes splenomegaly and hyperfunction, which accelerates red blood cell destruction and shortens the lifespan of red blood cells, which may promote the release of immature red blood cells and eventually lead to increased RDW^17,24,25^. These studies provide a theoretical basis for the correlation between blood routine indicators and liver fibrosis, but the magnitude of the correlation and the degree of liver function deterioration have not been clearly quantified, nor have they provided a predictable space for early liver fibrosis. Machine learning can make up for this deficiency. This study also find that age is also a key variable associated with liver fibrosis in *Schistosomiasis japonicum*, and the model predicts that the older the age, the greater the possibility of liver fibrosis. The significance of the machine learning method for this study lies in the establishment of a clinical prediction and identification model through simple blood routine indicators and patient age to give suggestions for the diagnosis of complex liver fibrosis.

This study built a machine learning model and evaluated the model by taking advantage of abundant data. Compared with the models mentioned in the published literature, this study only needs blood routine, age and gender to predict, providing clinicians with a more easy-to-operate and understandable diagnostic method.

But this study also has certain limitations. This study is a single-center retrospective study and some of the results discussed are also for an individual patient, which may not be able to avoid inherent selection bias and information bias. The next step of the study needs to conduct multi-center prospective research for external verification to further improve and promote this machine learning model. The variables of the current model only include the patient’s clinical information and test results. In order to optimize the performance of the identification model, the model can also include biomarkers from microbiome and metabolomics. However, at present, only using clinical variables can also reduce the burden on patients to a certain extent, and it has a certain degree of convenience in clinical application. Finally, the insufficient interpretability of SHAP values warrants the development of more understandable models in the future. In the future, we will further develop an automatic clinical scoring system based on nomograms or machine learning based on research data in order to provide clinicians with more practical and easy-to-understand tools.

## Methods

### Study population

The study population consisted of patients diagnosed with *Schistosoma japonicum* in Yueyang, Hunan Province, China. This city has historically been a high schistosomiasis epidemic area. Because it was located near Dongting Lake in the middle and lower reaches of the Yangtze River, where the Intermediate host *Oncomelania hupensis* breeds in large numbers.

*Schistosoma japonicum* infection was diagnosed according to the definition of Zhou et al.^26^. Including the following diagnostic criteria: life history in schistosomiasis-endemic areas, contact with infected water, specific schistosoma serology testing, color ultrasound, excreta (feces, urine) microscopic examination. Schistosomiasis infection was considered when schistosome ova were visualized in stool, urine or when the Schistosoma serology was positive.

Liver fibrosis was determined by ultrasound according to the World Health Organization diagnostic criteria for *Schistosoma japonicum* infection^27,28^. An experienced ultrasound expert divided the patients into two groups according to the ultrasound results: fibrosis group (with mesh-like changes and uneven hepatic echotexture); no-fibrosis group (without mesh-like changes, smooth and uniform hepatic echotexture). The diagnosis was double-checked by another experienced schistosomiasis specialist.

### Data collection

A retrospective medical record review was conducted from June 2019 to June 2022 at Xiangyue Hospital, Yueyang City, Hunan Province of China. All patients underwent blood tests and ultrasound evaluation at admission. All variables were extracted from the hospital’s electronic medical record system. The data include: patient demographic characteristics, blood routine indicators and other variables. KNN filling method is used to fill in the missing data. The principle is to identify k samples that are spatially similar or close in the data set through distance measurement, and then use these k samples to estimate the value of the missing data point. The percentage of missing data points is presented in Supplementary Table 5. The LassoCV method was used to screen out key variables. Data entry was performed by a full-time research physician or medical student. This study was conducted and approved by the Ethics Committee of the third Xiangya Hospital of Central South University (No: 21149) and has been carried out in accordance with the Code of Ethics of the World Medical Association (Declaration of Helsinki) for experiments. All methods were performed in accordance with the relevant guidelines and regulations. The need of informed consent was waived by the Ethics Committee of the third Xiangya Hospital of Central South University due to retrospective nature of the study. The privacy of all participants is fully protected.

### Feature selection

Patients were divided into hepatic fibrosis and non-hepatic fibrosis groups according to their color Doppler ultrasound results. Patients with hepatitis B virus (hepatitis B surface antigen seropositive), hepatitis C virus (HCV antibody seropositive), human immunodeficiency virus (HIV antibody seropositive), alcoholic and non-alcoholic fatty liver disease (ultrasound scanning and alcohol consumption above 30°g daily), decompensated liver disease or liver cancer (ultrasound and liver function tests), and organ transplantation (self-reported) were excluded. The key variables are selected by LassoCV method for subsequent modeling.

### Study design

First, the classification task was completed using 6 machine learning algorithms, including: ‘XGB Classifier’, ‘Logistic Regression’, ‘LightGBM Classifier’, ‘Random Forest Classifier’, ‘Support Vector Classification’, ‘K Neighbors Classifier’. Fivefold cross-validation method was used for validation. Each model was evaluated using AUC, clinical decision curve plot, accuracy, sensitivity, specificity, positive predictive value, negative predictive value, and F1 score. The ROC diagram and the forest diagram show the ROC results of each model for the prediction of “hepatic fibrosis”.

After selecting the best algorithm through multi-algorithm model comparison, the best algorithm was used to model again. Different from multi-model comparison, when using the best-performing algorithm for modeling, we randomly select 15% of the total samples as the test set, and the remaining samples are used as the training set for fivefold cross-validation.

### Model interpretation

The SHAP package in python can interpret the output of machine learning models, considering all features as “contributors”. For each prediction sample, the model will generate a prediction value, and its biggest advantage is that it can reflect the influence of the characteristics in each sample and show the positive and negative effects. This study used the SHAP package to interpret the model. SHAP value plots were used to show the contribution of each variable in the model. Model variable importance plots were used to show the importance ranking of each variable. Force diagrams were used to illustrate how each variable affects the predicted outcome for each sample with two examples.

### Statistical method

The python used in this study is version 3.7. The statsmodels 0.11.1 package in Python was used to count whether each variable was different between two groups of people. The analysis method was selected according to the distribution of samples, homogeneity of variance, and sample size. Chi-square test was used for categorical variables. Student’s t-test or Mann–Whitney U-test was used for quantitative variables.

In this study, LassoCV was used to screen key variables, and factors with a coefficient of 0 were automatically eliminated (sklearn 0.22.1 package in Python). Lasso obtains a more refined model by constructing a penalty function, so that it compresses some regression coefficients, that is, forces the sum of the absolute values of the coefficients to be less than a certain fixed value; at the same time, sets some regression coefficients to zero. Therefore, the advantage of subset shrinkage is preserved, and it is a biased estimate for dealing with data with multicollinearity. In the multi-model and best-model modeling process, the xgboost 1.2.1 package of Python is used for XGBoost algorithm modeling, the lightgbm 3.2.1 package of Python is used for LightGBM algorithm modeling, and the sklearn 0.22.1 package of Python was used to build other models. The shap 0.39.0 package in python was used to demonstrate the interpretability of the model.

### Ethical standards

Ethics approval was obtained from the Ethics Committee of the third Xiangya Hospital of Central South University.

### Supplementary Information

Supplementary Figures.

Supplementary Tables.

## Data Availability

The datasets used and/or analysed during the current study available from the corresponding author on reasonable request.
