# Supplementary figures and images for: Using blood routine indicators to establish a machine learning model for predicting liver fibrosis in patients with Schistosoma japonicum
Source: Sci Rep. 2024 May 20;14:11485. doi: 10.1038/s41598-024-62521-1 (PMC11106071; doi:10.1038/s41598-024-62521-1)

Supplementary Figure 1 Forest plot


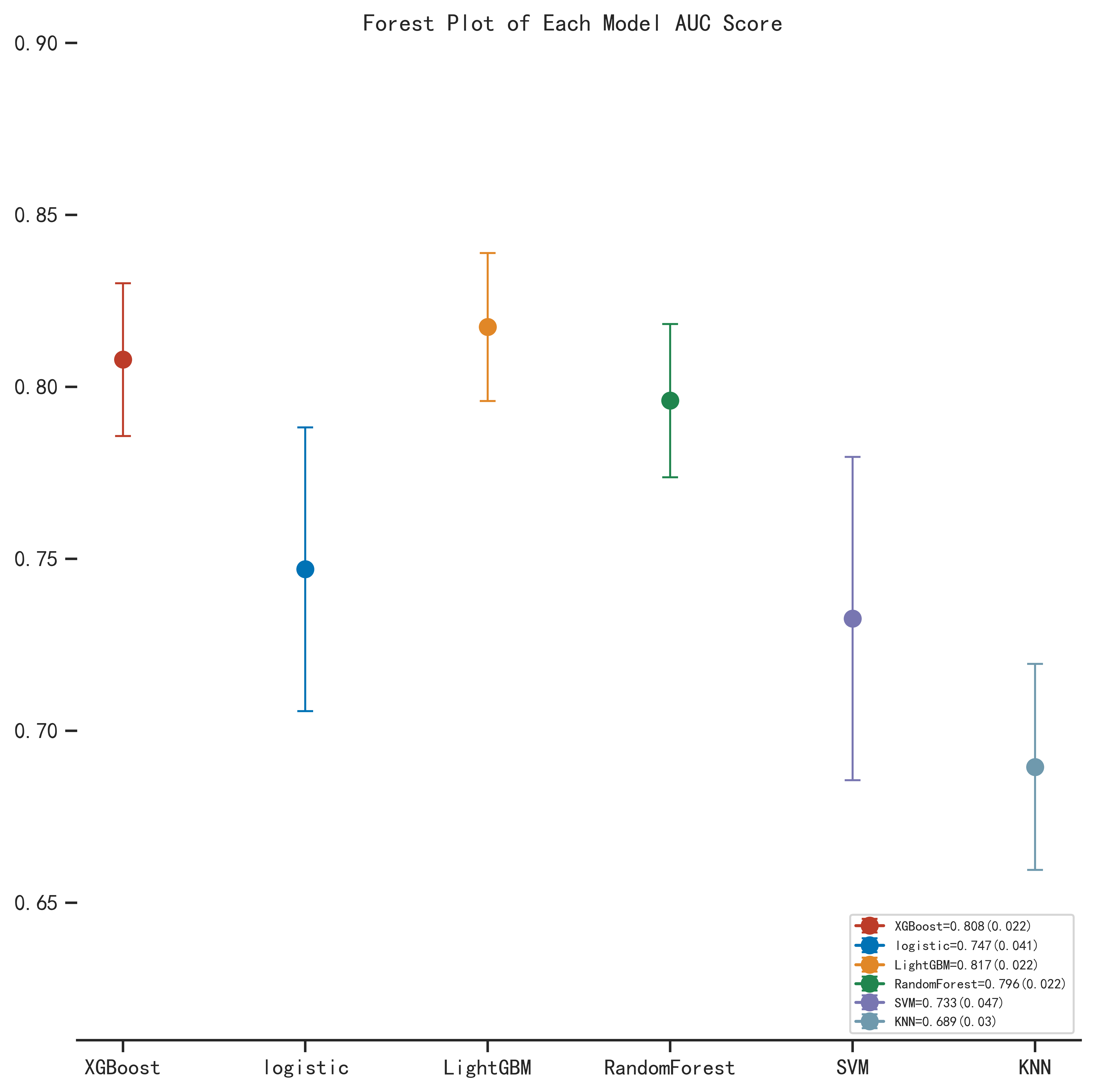


Supplementary Figure 2 Clinical decision curve


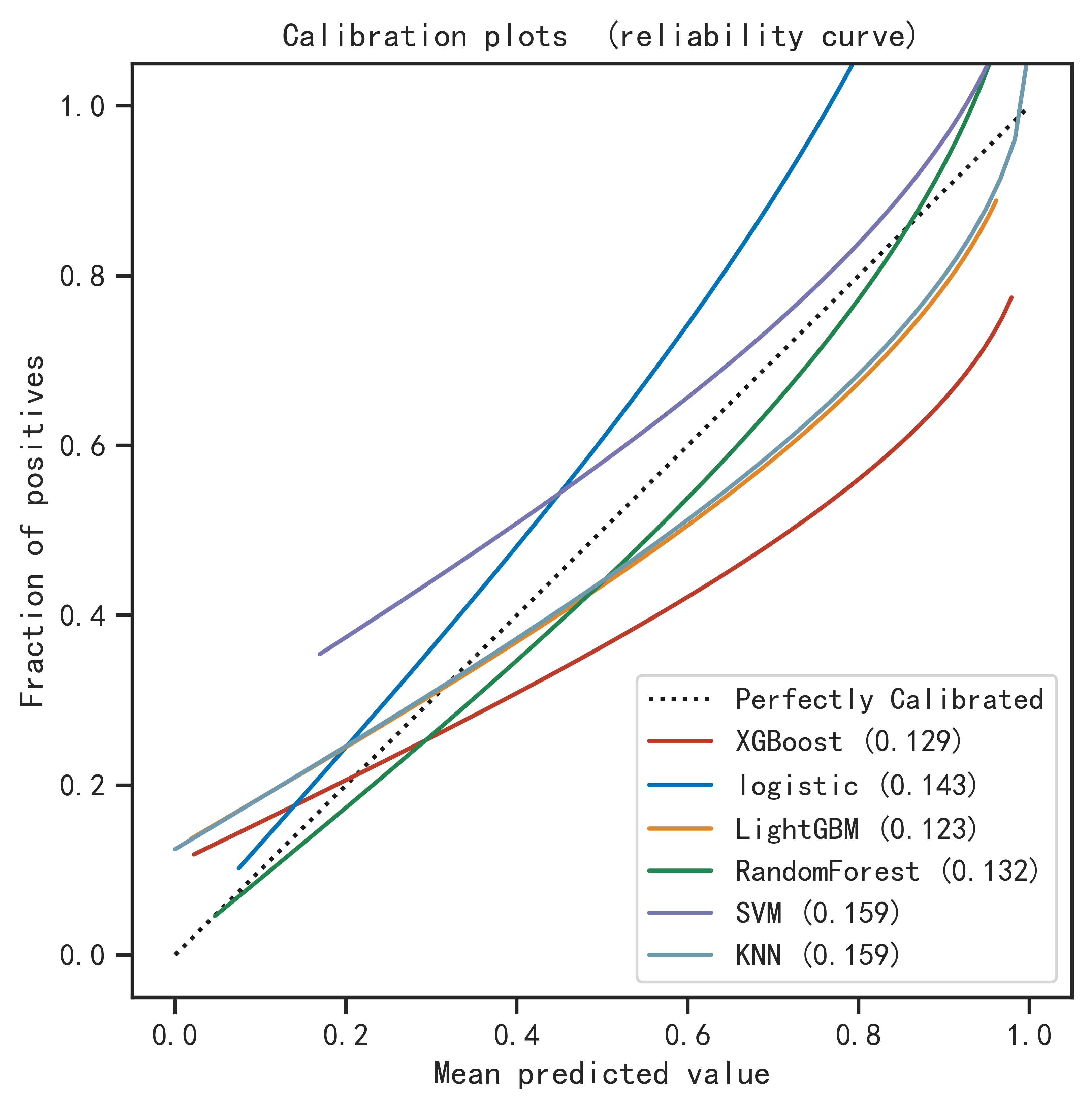

Supplement: Supplementary file 1 — Supplementary Figures. [file 41598_2024_62521_MOESM1_ESM.docx]
