# Supplementary material for: Using blood routine indicators to establish a machine learning model for predicting liver fibrosis in patients with Schistosoma japonicum
Source: Sci Rep. 2024 May 20;14:11485. doi: 10.1038/s41598-024-62521-1 (PMC11106071; doi:10.1038/s41598-024-62521-1)
Supplement: Supplementary file 2 — Supplementary Tables. [file 41598_2024_62521_MOESM2_ESM.docx]

**Supplementary Table 1 Multi-model classification-validation set results summary**

| **Model** | **AUC** | **Cut-off** | **Accuracy** | **Sensitivity** | **Specificity** | **Positive predictive value** | **Negative predictive value** | **F1 Score** | **Kappa** |
| --- | --- | --- | --- | --- | --- | --- | --- | --- | --- |
| XGBoost | 0.808(0.022) | 0.863(0.011) | 0.817(0.020) | 0.680(0.045) | 0.865(0.018) | 0.836(0.064) | 0.814(0.015) | 0.748(0.044) | 0.437(0.071) |
| logistic | 0.747(0.041) | 0.328(0.031) | 0.767(0.027) | 0.609(0.075) | 0.832(0.043) | 0.574(0.055) | 0.844(0.018) | 0.586(0.036) | 0.410(0.048) |
| LightGBM | 0.818(0.022) | 0.876(0.009) | 0.807(0.022) | 0.709(0.070) | 0.842(0.017) | 0.842(0.071) | 0.803(0.017) | 0.769(0.064) | 0.394(0.081) |
| RandomForest | 0.797(0.022) | 0.450(0.032) | 0.805(0.018) | 0.683(0.057) | 0.827(0.030) | 0.680(0.050) | 0.838(0.009) | 0.681(0.052) | 0.463(0.040) |
| SVM | 0.732(0.047) | 0.273(0.014) | 0.713(0.036) | 0.620(0.077) | 0.792(0.049) | 0.475(0.050) | 0.833(0.021) | 0.537(0.060) | 0.321(0.071) |
| KNN | 0.690(0.030) | 0.400(0.000) | 0.776(0.013) | 0.474(0.135) | 0.840(0.081) | 0.662(0.046) | 0.795(0.010) | 0.542(0.103) | 0.324(0.044) |

**Supplementary Table 2 Training set results**

| **AUC** | **Cut-off** | **Accuracy** | **Sensitivity** | **Specificity** | **Positive predictive value** | **Negative predictive value** | **F1 Score** |
| --- | --- | --- | --- | --- | --- | --- | --- |
| 1.000 (0.000) | 0.882 (0.010) | 0.999 (0.000) | 1.000 (0.000) | 1.000 (0.000) | 1.000 (0.000) | 0.998 (0.000) | 1.000 (0.000) |

**Supplementary Table 3 Validation set results**

| **AUC** | **Cut-off** | **Accuracy** | **Sensitivity** | **Specificity** | **Positive predictive value** | **Negative predictive value** | **F1 Score** |
| --- | --- | --- | --- | --- | --- | --- | --- |
| 0.804 (0.020) | 0.882 (0.010) | 0.813 (0.008) | 0.704 (0.047) | 0.820 (0.036) | 0.878 (0.073) | 0.806 (0.006) | 0.780 (0.042) |

**Supplementary Table 4 Test set results**

| **AUC** | **Cut-off** | **Accuracy** | **Sensitivity** | **Specificity** | **Positive predictive value** | **Negative predictive value** | **F1 Score** |
| --- | --- | --- | --- | --- | --- | --- | --- |
| 0.837 | 0.881 | 0.759 | 0.686 | 0.860 | 0.933 | 0.741 | 0.791 |

**Supplementary Table 5 The percentage of missing data points**

| Variable | **Total number** | **Mean** | **Median** | **25% quantile** | **75% quantile** | **Minimum value** | **Maximum value** |
| --- | --- | --- | --- | --- | --- | --- | --- |
| Age | 1049 | 60.41 | 62 | 51 | 71 | 20 | 91 |
| White blood cell | 1049 | 5.509 | 5.4 | 4.4 | 6.43 | 1.47 | 16.32 |
| Neutrophils | 1049 | 3.407 | 3.12 | 2.43 | 3.96 | 0.92 | 116 |
| Lymphocytes | 1049 | 1.692 | 1.65 | 1.24 | 2.07 | 0.2 | 13.4 |
| Monocytes | 1049 | 0.333 | 0.32 | 0.25 | 0.4 | 0.08 | 1.44 |
| Eosinophils | 1049 | 0.175 | 0.13 | 0.08 | 0.21 | 0 | 2.63 |
| Basophils | 1049 | 0.016 | 0.01 | 0.01 | 0.02 | 0 | 0.6 |
| Red blood cells | 1049 | 4.429 | 4.48 | 4.07 | 4.84 | 1.48 | 7.04 |
| HB | 1049 | 135.576 | 138 | 125 | 149 | 34 | 193 |
| HCT | 742 | 41.113 | 41.9 | 38.125 | 45 | 12.8 | 57.5 |
| MCV | 1049 | 93.752 | 94.5 | 90.6 | 98.2 | 33.2 | 118.3 |
| MCH | 1049 | 30.851 | 30.9 | 29.8 | 32.1 | 17.9 | 94.8 |
| MCHC | 1049 | 326.729 | 328 | 321 | 334 | 32.3 | 659 |
| RDW-SD | 742 | 44.848 | 44 | 41.6 | 46.8 | 34.4 | 73.2 |
| RDW-CV | 742 | 13.369 | 12.9 | 12.4 | 13.7 | 10.7 | 27.9 |
| Platelets | 1049 | 177.983 | 175 | 137 | 216 | 23 | 557 |
| Platelet volume | 732 | 0.194 | 0.19 | 0.16 | 0.23 | 0.03 | 0.61 |
| Platelet distribution width | 732 | 16.129 | 16.2 | 15.7 | 16.6 | 10.1 | 26.7 |
| Indirect bilirubin | 1049 | 10.17 | 9.3 | 7 | 12.5 | 1.2 | 98.6 |
| Albumin | 1049 | 42.863 | 44 | 40.6 | 46.1 | 13.5 | 53.7 |
| Globulin | 1049 | 26.671 | 26 | 23.8 | 28.7 | 14.3 | 44.6 |
| ALT | 1049 | 27.557 | 24 | 18 | 32 | 8 | 221 |
| AST | 1049 | 27.318 | 24 | 20 | 31 | 4 | 179 |
| Total bile acid | 741 | 7.034 | 3.6 | 2.2 | 7.2 | 0.2 | 206.7 |
| Alkaline phosphatase | 1027 | 82.459 | 74 | 60 | 92 | 7 | 633 |
| Glutamyl transpeptidase | 1027 | 53.556 | 29 | 19 | 53 | 5 | 1147 |
| Urea nitrogen | 1043 | 5.354 | 4.78 | 4.02 | 5.86 | 1.45 | 81.2 |
| Creatinine | 1043 | 70.923 | 67.2 | 56.15 | 77.8 | 14.6 | 653.3 |
| Uric | 1042 | 349.532 | 344 | 281.25 | 406.75 | 84 | 888 |
| Triglycerides | 1021 | 1.531 | 1.18 | 0.86 | 1.7 | 0.31 | 21.19 |
| Total cholesterol | 1025 | 4.917 | 4.85 | 4.21 | 5.55 | 1.18 | 9.73 |
| HDL | 1025 | 1.383 | 1.33 | 1.11 | 1.59 | 0.53 | 3.16 |
| LDL | 1026 | 3.257 | 3.22 | 2.58 | 3.87 | 0.87 | 7.05 |
| Glucose | 1032 | 5.686 | 5.32 | 4.93 | 5.85 | 3.72 | 24.9 |
| APTT | 332 | 26.493 | 25.25 | 22.775 | 28.9 | 16 | 49.9 |
| PT | 332 | 11.914 | 11.55 | 10.5 | 12.8 | 8.6 | 25.4 |
| INR | 332 | 1.085 | 1.055 | 0.95 | 1.17 | 0.77 | 2.33 |
| Prothrombin activity | 332 | 121.857 | 115.75 | 85.9 | 152.5 | 33 | 281.5 |
| Fibrinogen | 332 | 2.538 | 2.43 | 2.07 | 2.97 | 0.95 | 5.47 |
| TT | 332 | 19.107 | 18.7 | 17.7 | 19.8 | 14.4 | 107.7 |

Note: TT, Thrombin time; INR, International normalized ratio; PT, Prothrombin time; APTT, Activated partial thromboplastin time; LDL, Low-density lipoprotein; HDL, High-density lipoprotein; AST, Aspartate aminotransferase; ALT, Alanine aminotransferase; RDW-CV, Red cell distribution width-coefficient of variation count; RDW-SD, Red cell distribution width-standard deviation; MCHC, Mean corpuscular hemoglobin concentration; MCH, Mean corpuscular hemoglobin; MCV, Mean corpuscular volume; HCT, hematocrit; HB, Hemoglobin.
